# Comparing contamination rates of sterile-covered and uncovered transducers for ultrasound-guided peripheral intravenous lines

**DOI:** 10.1186/s13089-023-00347-0

**Published:** 2024-02-07

**Authors:** Yonathan Estrella, Nathan Panzlau, Kevin Vinokur, Samuel Ayala, Maya Lin, Theodore Gaeta, Lawrence Melniker, Gerardo Chiricolo, Nazey Gulec

**Affiliations:** 1 Department of Emergency Medicine, New York Presbyterian Brooklyn Methodist Hospital, Brooklyn, NY USA; 2 Department of Emergency Medicine, RWJBarnabas Health Community Medical Center, Tom’s River, NJ USA; 3 Emergency Care Specialists, Corewell Health, Michigan State University, East Lansing, MI USA; 4 Department of Emergency Medicine, Brookdale Hospital, Brooklyn, NY USA

**Keywords:** Ultrasound-guided procedures, Randomized control study, Hospital acquired infections, Intravenous catheters, Venous access

## Abstract

**Introduction:**

Physicians frequently use point-of-care ultrasound for intravenous access and bloodwork in the ED. Recently, AIUM and ACEP released recommendations on ultrasound-guided peripheral intravenous lines (USPIVs), but there are no agreed upon standardized policies. We sought to determine whether the use of sterile-covered transducers (SCT) decreases the rate of contamination when compared to uncovered transducers (UCT) after standard low-level disinfection (LLD).

**Methods:**

This is a randomized control trial comparing contamination rates of US transducers between SCT and UCT after their use for USPIV by the vascular access team, also known as the “PICC” team, over a 3-month period. A sample of admitted patient with an USPIV order were included and randomized to SCT (experimental) or UCT (control) arms. Transducers were swabbed and inserted into the SystemSURE Plus Adenosine Triphosphate (ATP) Luminometer to calculate Relative Light Units (RLU). We performed a cost analysis of requiring sterile covers for USPIVs.

**Results:**

The UCT and SCT arms contained 35 and 38 patients, respectively. The SCT group had a mean of 0.34 compared to the UCT group mean of 2.29. Each sterile cover costs $8.49, and over 3000 USPIVs are placed annually by the “PICC” team.

**Conclusion:**

Contamination rates were similar among the UCT and SCT groups after LLD. 254 inpatient USPIVs are performed monthly, not including failed attempts or covers used in the ED where USPIV placement is an essential part of ED workflow. This study suggests that the use of SCT does not significantly affect transducer contamination rates. These findings question burdensome regulatory hospital policies that are not evidence-based.

**Supplementary Information:**

The online version contains supplementary material available at 10.1186/s13089-023-00347-0.

## Introduction

The CDC estimates that one in every 31 hospitalized patients suffers from a Hospital Acquired Infection (HAI). In addition to the increase in morbidity and mortality placed on the individual patient, it costs the healthcare system billions of dollars each year [1]. It is debated whether ultrasound (US) transducers act as potential fomites or vectors for HAIs and if the use of sterile transducer covers help to reduce rates of contamination.

The Food and Drug Administration has upheld the same standards since 1957 which called for high-level disinfection (HLD) in addition to the use of sterile gel and sterile transducer covers [2]. This was echoed by The European Society of Radiology Ultrasound Working Group [3]. As of March 2021, the American Institute of Ultrasound in Medicine (AIUM) guidelines stated that USPIV placement is a “clean procedure requiring non-sterile transducer covers” [4]. In April 2021, the American College of Emergency Physicians (ACEP) released a statement declaring that “probes used externally for percutaneous procedures should be covered with single-use protective covers and sterile gel applied. They should subsequently be cleaned using low-level disinfection”. The statement was referring to “single-use sterile probe covers matching the sterility of the procedure” [5]. In the case of USPIVs, operators are not required to wear PPE or sterile drapes as they would when performing a central line, thoracentesis, or paracentesis. They typically only use non-sterile gloves. This again brings into question the need for sterile transducer covers. ACEP also characterized protective barriers such as medical gloves, condoms, and adhesive barriers as being of acceptable quality.

Low-level disinfection (LLD) refers to the use of chemicals to destroy bacteria (with the exception of tubercle bacilli) and most viruses, which can be achieved using various disposable wipes [6–9]. Theses guidelines are supported by the Association for Professionals in Infection Control and Prevention (APIC) as well as the Society for Healthcare Epidemiology of America (SHEA). High-level disinfection refers to the use of chemical sterilants, germicides, or hydrogen peroxide to completely eliminate all microorganisms and spores. For example, vaporized hydrogen peroxide is used in the Trophon EPR system as it has been shown to eradicate cancer causing strains of Human Papilloma Virus.

Although several governing bodies may agree, there is not much evidence available to support these policies. Furthermore, there are differing opinions on whether adhesive barriers and sterile film dressings are interchangeable with traditional sterile sleeve covers. Some experts may argue that the purpose of transducer covers is to avoid cross contamination between patients when using the same transducer on multiple subjects as opposed to preventing bodily fluid from coming into contact with the transducer [10]. Regardless of recommendations, a survey conducted in 2018 by Carrico et al. showed that ED practitioners are poorly adherent and typically perform ultrasound-guided peripheral intravenous line (USPIV) placement under non-sterile conditions [11].

The objective of this study was to determine if contamination rates differed between groups randomized to uncovered ultrasound transducers and transducers covered with sterile barriers following low-level disinfection. We also sought to calculate the amount of money saved if we no longer used sterile transducer covers when placing USPIVs.

## Methods

This is a randomized control trial comparing rates of contamination between sterile-covered (SCT) and uncovered transducers (UCT) after their use for USPIV placement by a member of the vascular access team at NYP-BMH over a 3-month period in 2019. The hospital employs a team of vascular access specialists who place peripherally inserted central catheters (PICC) on patients who will require IV medications upon discharge. They also assist with obtaining peripheral access when the nursing and physician staff is unsuccessful. A 2015 SonoSite NanoMaxx Ultrasound System 1203 and L25 linear array 6-13 MHz transducer was utilized throughout the study. Sterile CIVCO 610–542 CIV-Flex transducer covers (14 × 91.5 cm) and sterile, single-use Aquasonic hypoallergenic, bacteriostatic, non-irritating gel packets were used.

This study included admitted patients with an USPIV electronic order when our primary investigator (PI) was available. These orders were only placed on admitted patients who were deemed to have difficult access after several failed attempts by the nursing and physician staff. Rstanarm version 2.21.3 package of R version 4.2.1 was used to perform a power calculation and determined the need for 35 subjects in each group [12]. Patients were randomized to the UCT (control) arm or SCT (experimental) arm using a standard randomization table. After successful USPIV placement and removal of transducer cover in the SPC group, all transducers were wiped with a single dry towel followed by a single 55% isopropyl alcohol cloth [8]. Transducers were then allowed to dry for 2 min based on the manufacturer’s recommendations for required contact time [8]. At this point, the transducer was swabbed and considered ready for its next use.

Transducers were swabbed with proprietary UltraSnap Surface Adenosine Triphosphate (ATP) monitoring swabs, and the samples were inserted into the Hygiena SystemSURE Plus ATP Monitoring Luminometer [13]. ATP monitoring is designed to measure residual organic matter. Any ATP that is picked up by the swab undergoes a reaction catalyzed by the enzyme luciferase. This reaction produces adenosine monophosphate (AMP) and energy emitted in the form of light which is detected by the luminometer. Results were available after 60 seconds in the form of relative light units (RLU) [14].

ATP bioluminescence is a rapid technique that is widely used to detect microbial contamination of food, food processing equipment, and water humidifiers [15–17]. Its use is becoming more popular in the medical field, such as validating cleaning practices of surgical instruments and endoscopes [18–21]. Several studies have shown good agreement with conventional microbiological culture methods and positive correlations with colony-forming units [22–25].

The cutoff RLU value for what is considered “clean” is set forth by the product manufacturer and varies across countries [26]. The Hygiena ATP System used in this study determines cleanliness with a RLU value less than 100 [27]. This study maintained that a RLU value less than 25 is considered clean. The protocol, device, and reference values used fall in line with guidelines set forth by our hospital’s Infection Control Department.

RLU means, medians, and ranges were calculated using Microsoft Excel (Microsoft Office, Microsoft Corporation, Redmond, WA, USA). A cost analysis was performed to assess the financial implications of requiring sterile transducer covers for USPIV placement, including tunneled central lines, midline catheters, and peripheral IVs. The average cost per sterile transducer cover and the number of USPIVs placed by the PICC team was used to calculate the financial burden. This study was approved by the IRB with a waiver of informed consent considering the PICC team routinely places IV lines without the use of a transducer cover. Patient identifiers were not included.

## Results

**Fig. 1:**
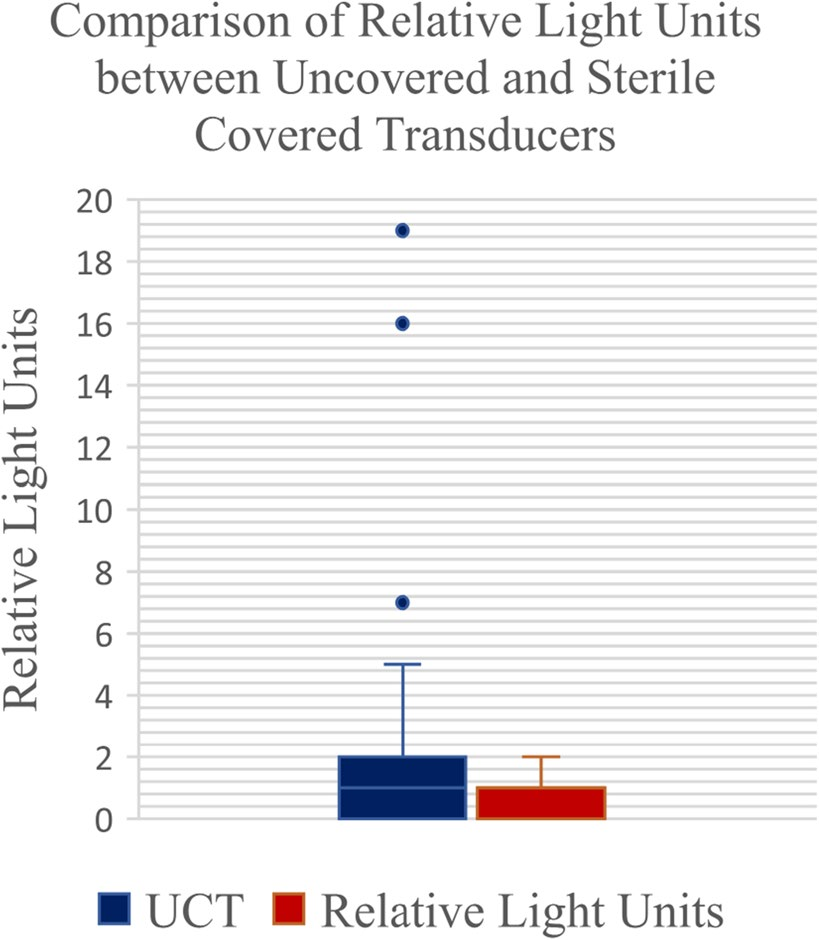
Box and whisker plot displaying relative light units among uncovered transducer (control) and sterile-covered transducer

73 patients were enrolled in this study. The control (UCT) arm had 35 samples with a mean RLU of 2.29. The experimental (SPC) arm had 38 samples with a mean RLU of 0.34 (Table 1). The difference between the two groups was not statistically significant (p = 0.006). This study had a statistical power of 0.82 assuming a test level of 0.05. Three outliers were identified in the control arm which were still included in the calculation (Fig. 1). Each sterile transducer costs the hospital $8.49. The PICC team averages 254 successful USPIVs per month. The incremental cost of requiring sterile transducer covers for USPIVs was estimated to be at least $25,877 annually.

**Table 1:** Statistical analysis comparing UCT and SCT groups

Relative light units	Sterile-covered transducer	Uncovered transducer
Median	0	1
Range	0–2	0–19
Mean	0.34	2.29
Variance	0.35	18.2
Standard deviation	0.58	4.27

## Discussion

SCTs use did not result in a statistically significant reduction in contamination rates in comparison to UCTs. Rates in both groups were below thresholds defined as “clean” by our local institution which resulted in cost savings. USPIV placement has been a major part of point-of-care ultrasound in the Emergency Department. About 12 million USPIVs are placed annually in North American [28]. Au, Arthur K, et al. found that USPIVs reduce the need for central lines by 80% and allow ED physicians to obtain intravascular access when traditional, landmark-guided attempts have failed [29]. Shokoohi et al. published similar findings in addition to high patient satisfaction [30, 31]. USPIV placement in the ED is common, and it will only increase in frequency considering its use is becoming more widespread among the nursing community. As US becomes more available, it is possible that it may become standard of care in order to minimize failed attempts and multiple needlesticks.

PICC lines are typically placed in a peripheral vein along the proximal arm and the end of the catheter lies in a larger vein such as the subclavian or superior vena cava. Midline catheters are also placed in the upper arm. The distal end remains in a peripheral vein but in very close proximity to a central vein. Both catheters remain in place for a much longer time when compared to PIVs which may increase the rates of catheter-related bloodstream infections. Because of this, it is necessary to follow strict sterile procedures. PIVs are typically replaced after 72–96 h which decreases the concern for seeding infection.

Adhikari et al. conducted a retrospective study in 2010 comparing infection rates between patients who received a traditional PIV and those who received a PIV with ultrasound guidance. In both groups, nurses placed the PIV lines and bacteriostatic lubricant was used. The US group used a non-sterile glove for barrier protection. Adhikari found no statistically significant difference in infection rates between the two groups [32].

Reisenauer et al. searched two independent institutional databases to identify rates of breast infections in patients who had underwent US-guided interventions. They included 12,708 patients who had undergone US-guided biopsies or aspirations. Investigators found a procedure-related infection incidence of 0.11%. Those 14 cases had localized soft tissue infections that were treated with oral antibiotics. There were no adverse events as defined by need for IV antibiotics, percutaneous intervention, surgical intervention, or hospitalization. Transducer covers were not used, but all of the transducers underwent intermediate-level disinfection. AIUM currently recommends using sterile transducer covers when performing US-guided breast biopsies. This study shows the risk of infection without the use of a transducer cover is extremely low, and the infections that do occur are easily treated [33].

Chu et al. seeded non-endocavitary transducers with increasing concentrations of MRSA to evaluate the efficacy of their institutional policy of cleaning these transducers with 0.5% accelerated hydrogen peroxide. They included concentrations of 10^4^, 10^5^, and 10^6^ CFU/mL to simulate typical bacterial loads on human skin in addition to higher concentrations of 10^7^, 10^8^, and 10^9^ CFU/mL. Transducers were swabbed prior to cleaning to ensure the transducers were successfully seeded. After cleaning, zero transducers grew MRSA, implying that 0.5% accelerated hydrogen peroxide was adequate for proper disinfection [34].

Similar to our study, the current literature does not support the use of sterile barriers when performing simple procedures under ultrasound guidance. While these studies suggest a transducer cover may be unnecessary, there were no studies to date specifically documenting a cost analysis. The policies created by AIUM and ACEP are meant to be guidelines and are subject to change as more literature becomes available. Each hospital has different infection control policies. NYP-Brooklyn Methodist Hospital maintains a policy that sterile transducer covers are not required when placing USPIVs. Changing our policy to meet AIUM and ACEP standards could increase hospital costs without a proven benefit in term of procedure cleanliness. Although the calculated cost of purchasing sterile transducer covers is not substantial, it is grossly underestimated considering it only accounts for successful USPIVs placed by the PICC team. It does not take into account failed attempts or the number of covers used by other providers throughout the hospital. The cost analysis was performed to get a sense of how much money a medical facility would have to absorb to provide the equipment as this would be more applicable in areas with limited resources in other parts of the world.

## Limitations

The three outliers in the control group were attributed to protocol violations; however, they were still clean by definition given that the RLU values were less than 25. Despite the three outliers, the remainder of the results were consistent, and the study has a statistical power of 0.82. The outliers were still included in the analysis as they did not change the outcome of the study. The outliers were due to protocol violations which conveniently emulate a real-life scenario where human error is expected.

This study was conducted by a PICC team nurse on patients who were already admitted to the hospital. Several patients included in the study were on contact or droplet precautions; however, this information was not recorded considering this information is not readily available outside of the inpatient setting. The same practices and protocols were carried out in addition to the use appropriate personal protective equipment. Additionally, isolation status did not interfere with the PICC team’s ability to place IV lines at this hospital.

RLUs were used to assess contamination as opposed to more validated methods such as bacterial growth on culture plates for various reasons. Using RLUs allowed us to obtain results in a timelier fashion. Secondly, ATP swabs were discarded after analysis, thus eliminating the concern for physical space constraints or the availability of an incubator with optimal growing conditions.

This study was conducted on patients admitted to the general medical floor. The ED is a less controlled environment and operator dependency plays a larger role; however, this is likely not a limiting factor considering identical disinfection practices are utilized in both scenarios. A subsequent study looking at covered versus uncovered USPIVs solely in the emergency department could be beneficial to support this claim.

Transducers were swabbed directly after the disinfection process to assess for contamination. The same transducer was then used on the next patient without additional manipulation. It is possible that the transducer could acquire fomites during transport between patient rooms or in the storage area. With this in mind, the cleaning process should be done prior to performing the procedure. This should not compromise the significance of the results. Additionally, while this study proves that LLD is effective in “cleaning” transducers, it also suggests that the use of sterile covers is redundant.

This study did not include patient comorbidities, immunocompetency, presence of concomitant infection, transmission-based precautions or subsequent culture results from potential downstream infections that may have resulted during a patient’s hospitalization. This was a biometric study that did not focus on patient-centered outcomes.

### Supplementary Information

**Additional file 1.** Relative Light UnitData for Sterile-covered and Uncovered Transducers.

## Data Availability

All data generated or analyzed during this study are included in this published article [and its Additional file 1] [35].

## Abbreviations

US: Ultrasound
PIV: Peripheral intravenous line
HAI: Hospital acquired infection
SCT: Sterile-covered transducers
UCT: Uncovered transducers
LLD: Low-level disinfection
HLD: High-level disinfection
RLU: Relative light units
PICC: Peripherally inserted central catheter
